# Triangular Lunulae in Papillon–Lefèvre Syndrome: A Case Report

**DOI:** 10.1002/ccr3.70339

**Published:** 2025-04-14

**Authors:** Zarak Khan Shiraz, Fahad Faizullah, Qaisar Ali Khan, Irfan Ullah, Mehran Khan, Bader Semakieh, Zain Ul Islam, Ravina Verma

**Affiliations:** ^1^ Khyber Teaching Hospital MTI KTH Peshawar Pakistan; ^2^ Hayatabad Medical Complex Peshawar Pakistan; ^3^ Arkansas College of Osteopathic Medicine Fort Smith Arkansas USA; ^4^ KMU Institute of Dental Sciences Kohat Pakistan; ^5^ St. George's University School of Medicine True Blue Grenada

**Keywords:** case report, genetic disorders, Nail–Patella syndrome, Papillon–Lefèvre syndrome, triangular lunulae

## Abstract

Triangular lunulae, typically associated with Nail–Patella Syndrome, may also present in other syndromes, as seen in this case. Papillon–Lefèvre syndrome is a rare genodermatosis linked to significant morbidity. Consanguinity is a recognized high‐risk factor. Early diagnosis and treatment can reduce morbidity in affected individuals, and raising societal awareness about consanguinity can help alleviate the disease's burden in populations where this practice is prevalent.

## 1 Introduction

First described by Papillon and Lefèvre in 1924 [1], Papillon–Lefèvre Syndrome (PLS) is a rare, autosomal recessive disorder [2] characterized by the presence of palmoplantar hyperkeratosis and frequent bacterial infections, particularly severe periodontitis, resulting in early loss of deciduous and, eventually, permanent teeth [2, 3]. The prevalence of PLS is reported to be 1–4 cases per million individuals [4]. Skin changes are the earliest to appear, usually within the first year of life, with diffuse, erythematous, palmoplantar hyperkeratosis being the most commonly noted presentation [3]. Additional features may include dural and choroid plexus calcifications, hyperkeratotic plaques on the elbows and knees, pseudo‐ainhums around digits, and infrequently, mental retardation [3, 4]. PLS is caused by a mutation in the CTSC gene encoding a lysosomal protease, cathepsin C [5]. The gene is widely and strongly expressed in immune cells, such as neutrophils and macrophages, and also at sites mainly affected in PLS, including the palms, soles, knees, and keratinized gingiva in the oral cavity, elucidating the mechanisms underlying the symptomatology that is observed [5]. Aberrations in the normal functioning of cathepsin C in neutrophils in PLS have been noted to result in their excessive recruitment to periodontal tissue which, paired with their reduced antimicrobial ability due to the protease deficiency, leads to the classical, severe periodontitis that is noted in the disorder and which is the main reason for the premature exfoliation of primary and secondary teeth [6]. Besides periodontitis, infections of other sites are also common in patients with PLS, including pyogenic skin infections, abscesses in internal organs such as the kidneys and liver, and recurrent respiratory and urinary tract infections [7]. An allelic variant of PLS, Haim–Munk syndrome, is also characterized by the cardinal features of PLS, namely, palmoplantar hyperkeratosis and premature periodontal destruction, but with the addition of onychogryphosis, acro‐osteolysis, and arachnodactyly [8].

Nail and Patella Syndrome is a distinct clinical entity, a rare autosomal dominant condition comprising variable nail, skeletal, and systemic anomalies, including patellar hypoplasia and agenesis, elbow dysplasia, radial head dislocation, characteristic iliac horns, nail abnormalities, nephropathy, and glaucoma [9]. Although nail abnormalities are variable, triangular lunulae in the fingernails are considered highly characteristic for the condition [10]. So far, the presentation of triangular lunulae has not been reported with other medical conditions. Identifying the feature in our patient with PLS is the first report of its nature.

## 2 Case History and Clinical Examination

A 16‐year‐old girl, the second eldest of four daughters to parents who were first‐degree cousins, was referred to the dermatology clinic at Khyber Teaching Hospital, Peshawar, Pakistan, with a 3‐month‐long complaint of a small, soft outgrowth from the lateral periungual skin fold of the right big toe that appeared spontaneously as an erythematous papule and grew slowly in size with time, eroding the adjacent nail plate and ultimately exuding purulent discharge. On further inquiry, the patient's mother reported a history of persistent and progressively worsening thickening, scaling, and fissuring of the palms and soles that was first noticed when the patient started walking at the age of one. Initially, the complaint was restricted to the palms and soles but later progressed to involve the dorsal surfaces of the hands and feet. The skin complaints were accompanied by frequent swelling and friability of the gums, which became more noticeable and pronounced when the patient began to prematurely lose her primary dentition at the age of two, which had sprouted normally. The gum complaints subsided once all deciduous teeth were lost, only to reappear with the eruption of secondary dentition.

Consequently, the patient also lost most of her secondary dentition by the age of 11. The patient also had a history of recurrent, focal skin infections over the years, primarily on the scalp, which were slow to resolve and would frequently lead to ulceration. However, these lesions were responsive to topical and oral antibiotics. The patient was treated with oral amoxicillin and metronidazole for skin infection and periodontitis, as sensitivity was shown for these two antibiotics. There was also a complaint of progressively worsening dystrophy of the nails of the fingers and toes. However, the patient denied any history of acute trauma, repetitive trauma by self, manicures, or any other manipulation that could have contributed to the nail changes. Additionally, there was a history of the development of thickened, scaly plaques on the elbows and knees that had resolved a few years back to leave behind atrophic, hypopigmented skin. According to the mother, there were no ante‐ or postnatal complications, and the patient was otherwise healthy and achieved her developmental milestones on time. Over the years, the patient had had numerous outpatient visits to dental and dermatological clinics; however, a firm diagnosis was not made, resulting in interrupted and mostly symptomatic medical management. The youngest sister of the patient, aged 9 years, was also found to be following an almost identical sequence of symptoms as the patient, including palmoplantar thickening, loss of secondary dentition, and frequent slow‐healing, scalp infections.

**FIGURE 1:**
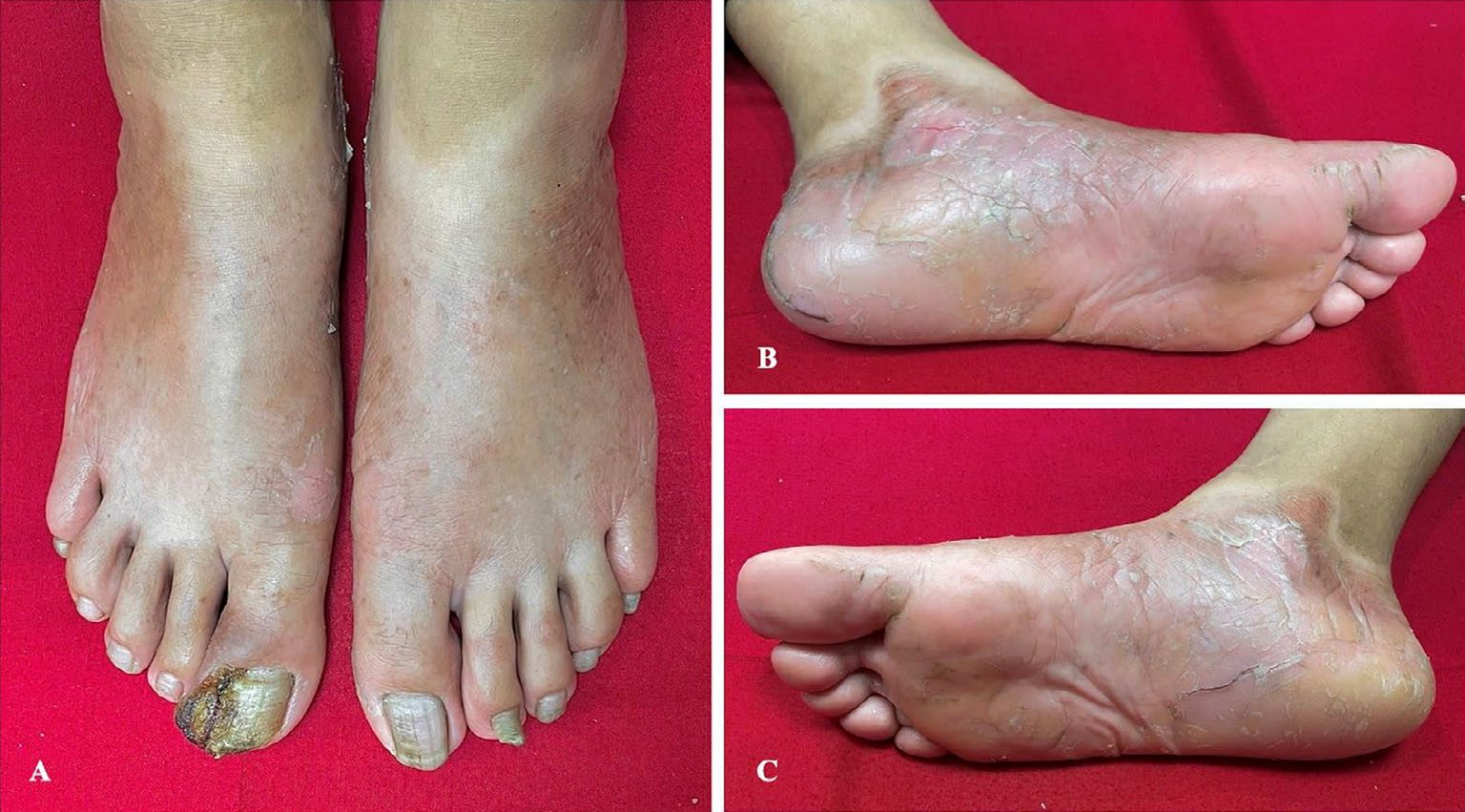
Diffuse plantar erythema, thickening, and scaling (B and C) extend onto the dorsum of the feet (A).

**FIGURE 2:**
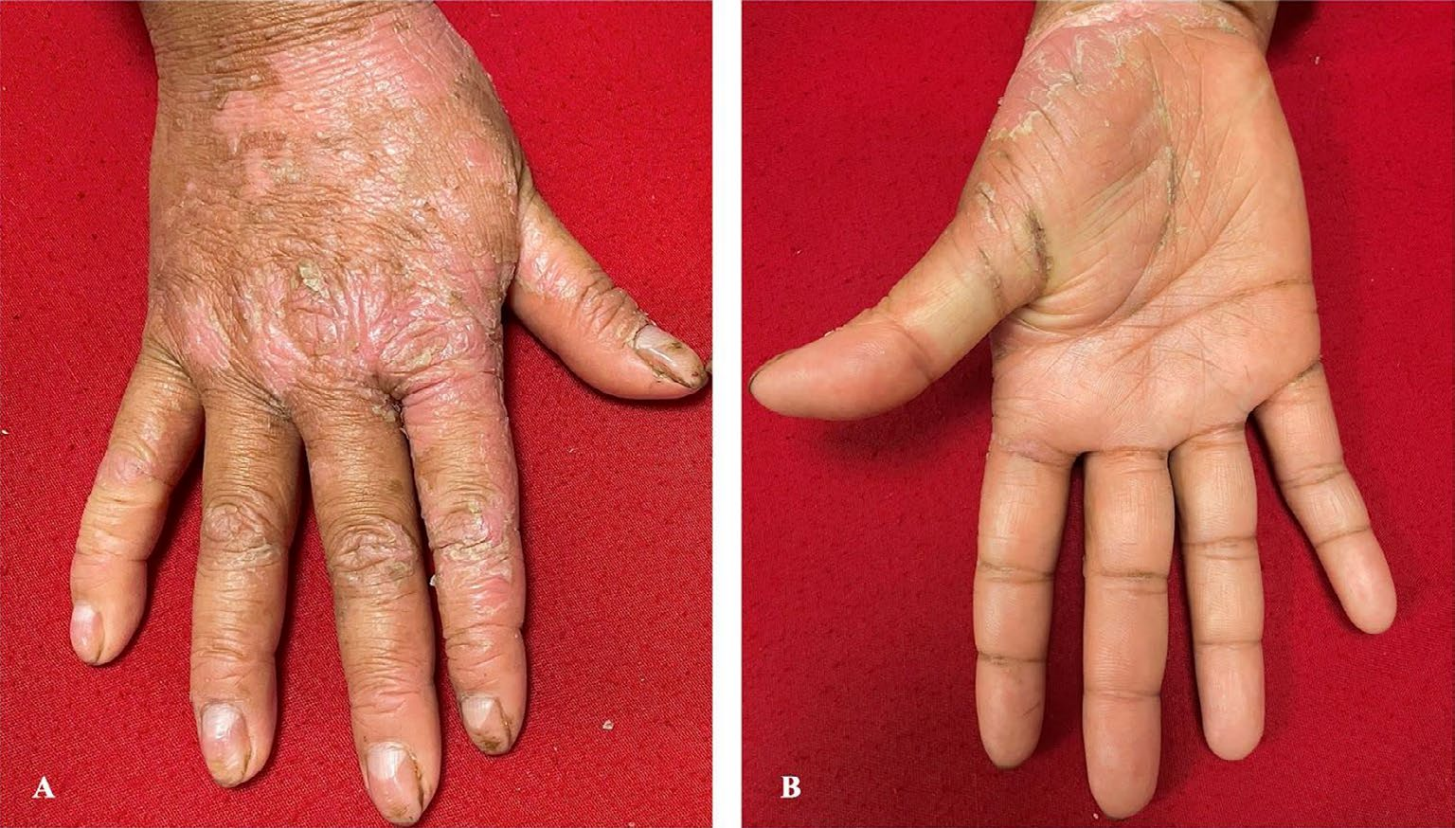
Diffuse palmar thickening, with erythema and scaling on the wrist and thenar eminence of the right hand (B) extending onto the dorsum of the hand (A). Similar changes were noted on the left hand.

On examination, the patient had diffuse palmoplantar erythema, thickening, and scaling that extended confluently onto the dorsal aspects of the hands and feet. Multiple areas of fissuring were noted on the palms and soles (Figures 1 & 2).

**FIGURE 3:**
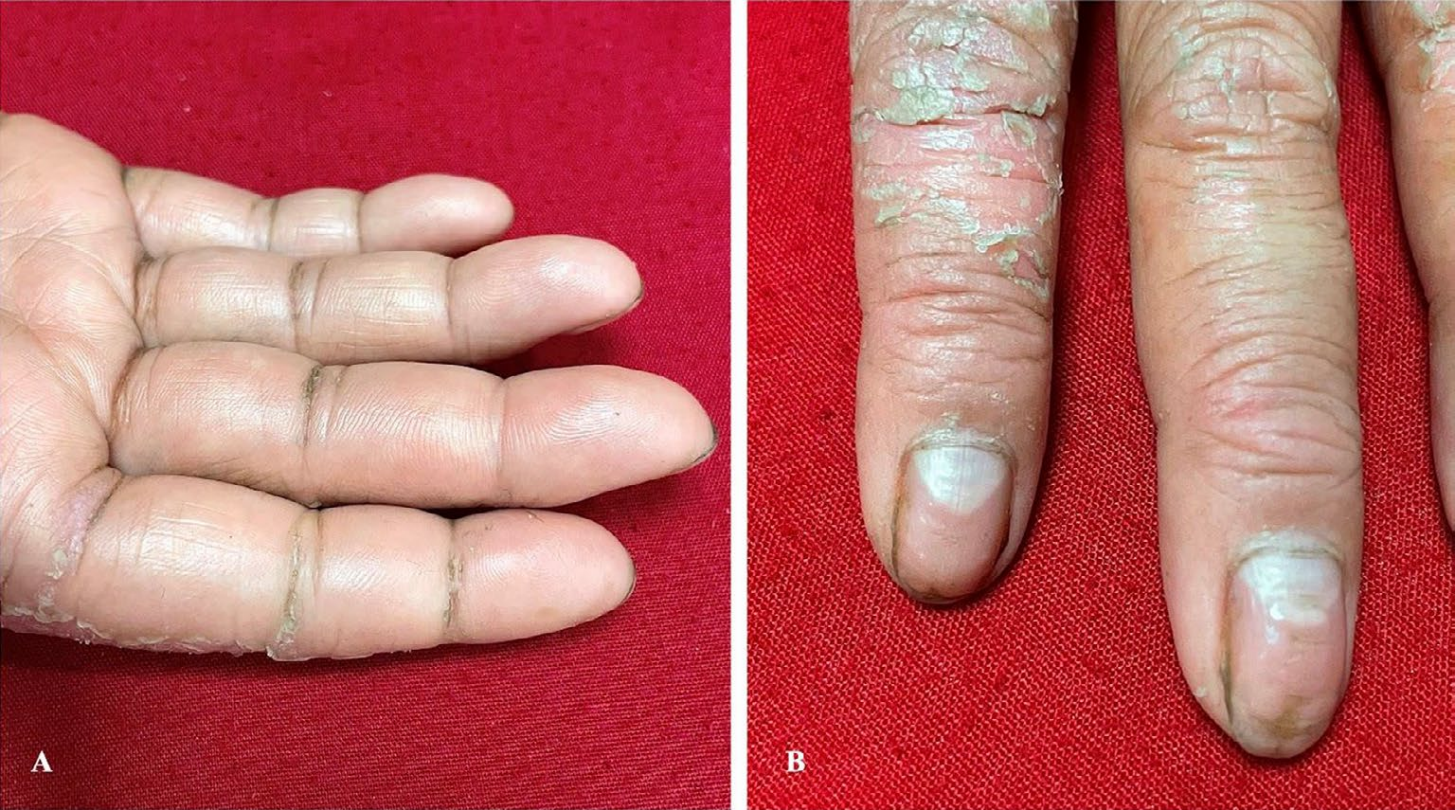
(A) Early pseudo‐ainhum formation over the interphalangeal joints. (B) Exaggerated longitudinal curvature of the fingernails along with pits and horizontal grooves. A triangular lunula is also noted.

Concentric band‐like thickening of the skin of the fingers was also observed (Figure 3A), resulting in accentuation of the pulp of the soft tissue between the interphalangeal joints. The fingernails showed an exaggerated longitudinal curvature, an enlarged triangular lunula (Figures 2A & 3B), multiple pits and horizontal grooves, and slight distal discoloration (Figure 3B).

**FIGURE 4:**
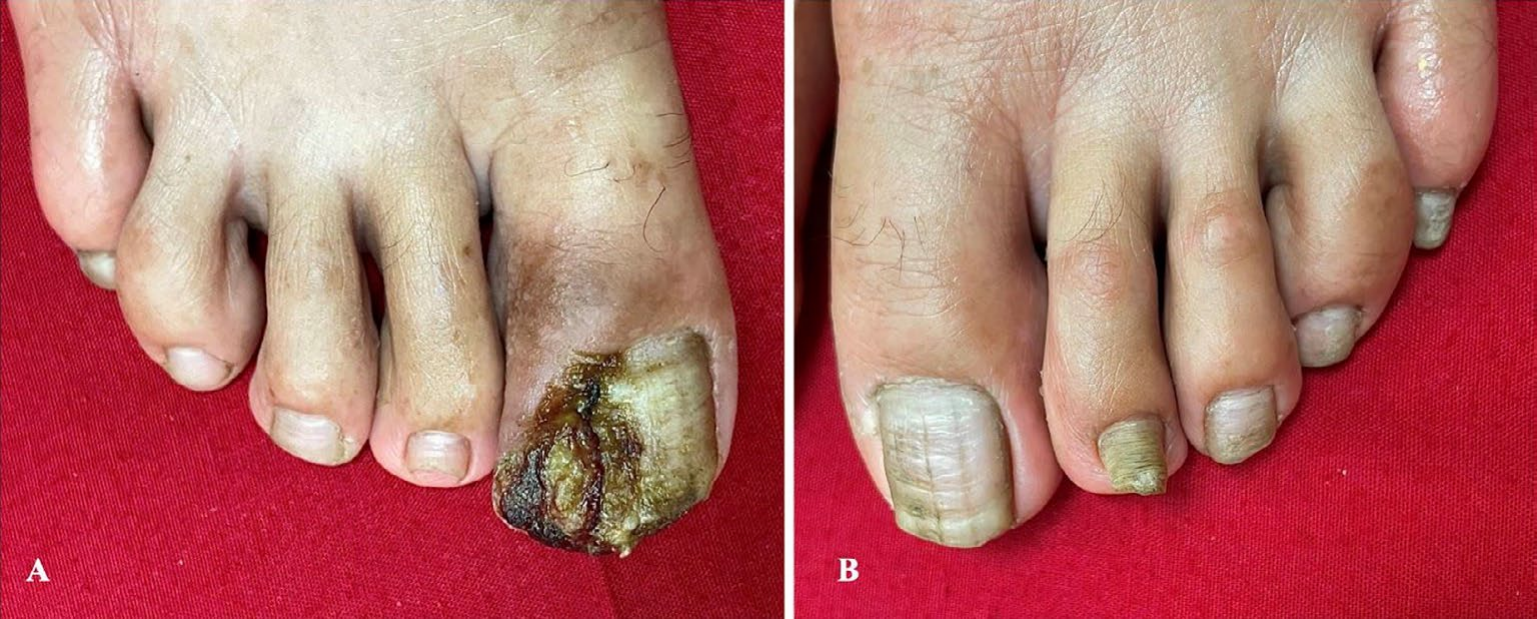
(A) An exophytic outgrowth from the right toe, likely indicating granulation tissue post‐pyogenic site infection. (B) Onychodystrophy with prominent onychogryphosis of the second toenail of the left foot.

The right big toenail had a 1 × 1 cm soft, fluctuant, exophytic growth with purulent exudate and crusting, arising from the left, lateral periungual skin fold and involving the lateral nail bed (Figure 4A). Both big‐toe nails showed yellow discoloration and multiple horizontal and longitudinal grooves. The second and fifth toenails of the left foot showed onychogryphosis, while the remainder of the toenails of the left foot showed mild dystrophy (Figure 4B). The second to fifth toenails of the right foot were normal (Figure 4A). Triangular lunulae were not noted in the toenails.

**FIGURE 5:**
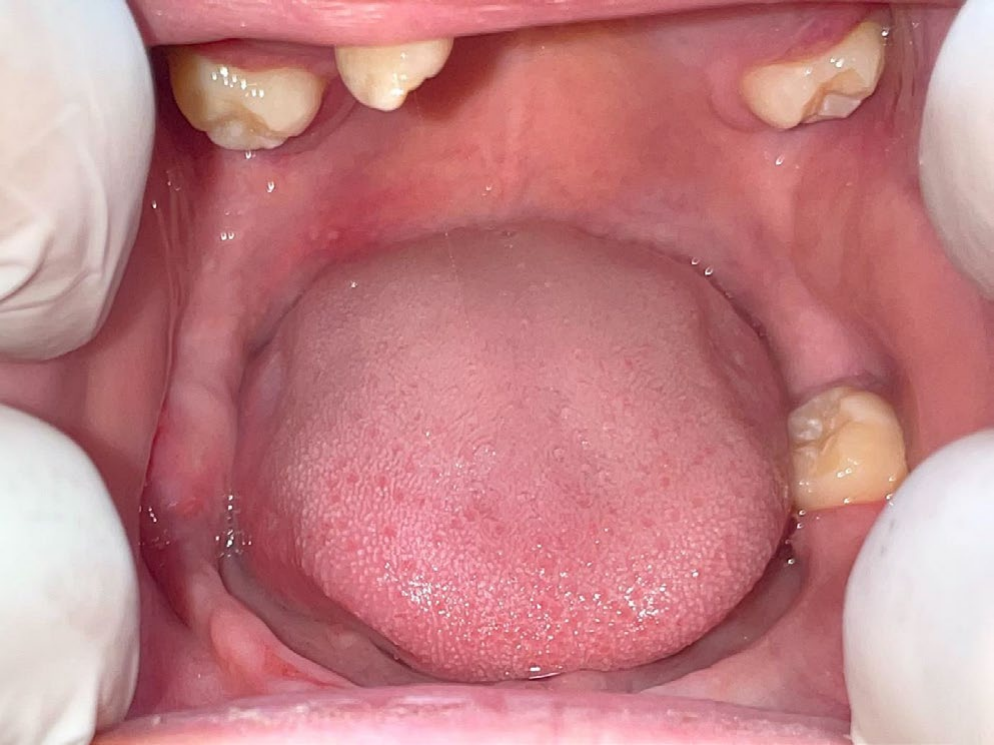
Loss of multiple secondary dentition with some inflammation of right lower gingival tissue noted.

**FIGURE 6:**
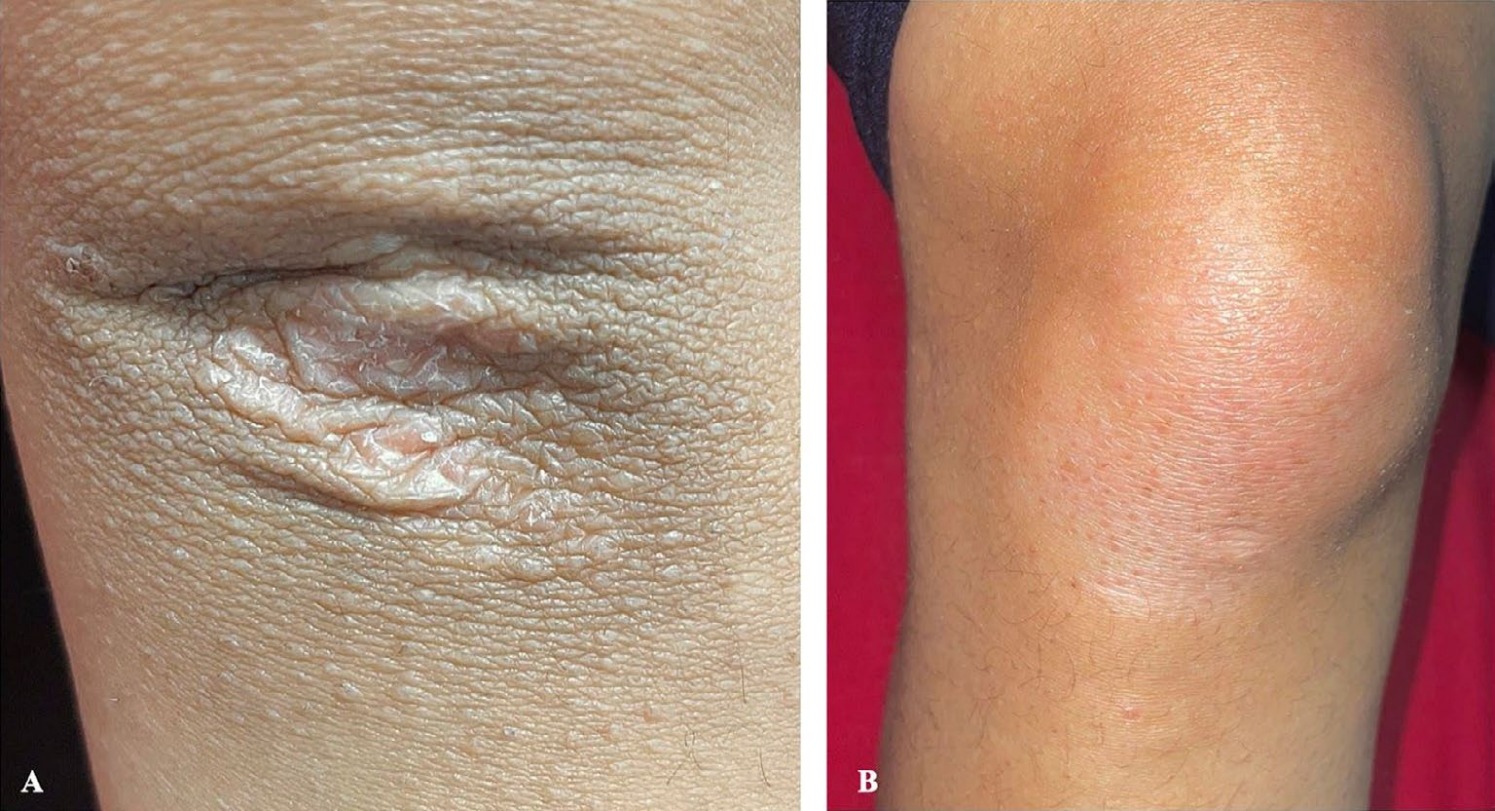
(A) Hypopigmented, atrophic plaque on right elbow. (B) Hypopigmented patch on the right knee.

Oral cavity examination revealed signs of mild gingival inflammation of the right lower side. Two upper molars, a single left lower molar, and a loose right upper incisor were the only teeth noted (Figure 5). The elbows had bilaterally symmetrical hypopigmented, atrophic plaques (Figure 6A), whereas the knees showed similar plaques without atrophy (Figure 6B). No abnormality of the hair was noted. A systemic examination was also unremarkable.

**FIGURE 7:**
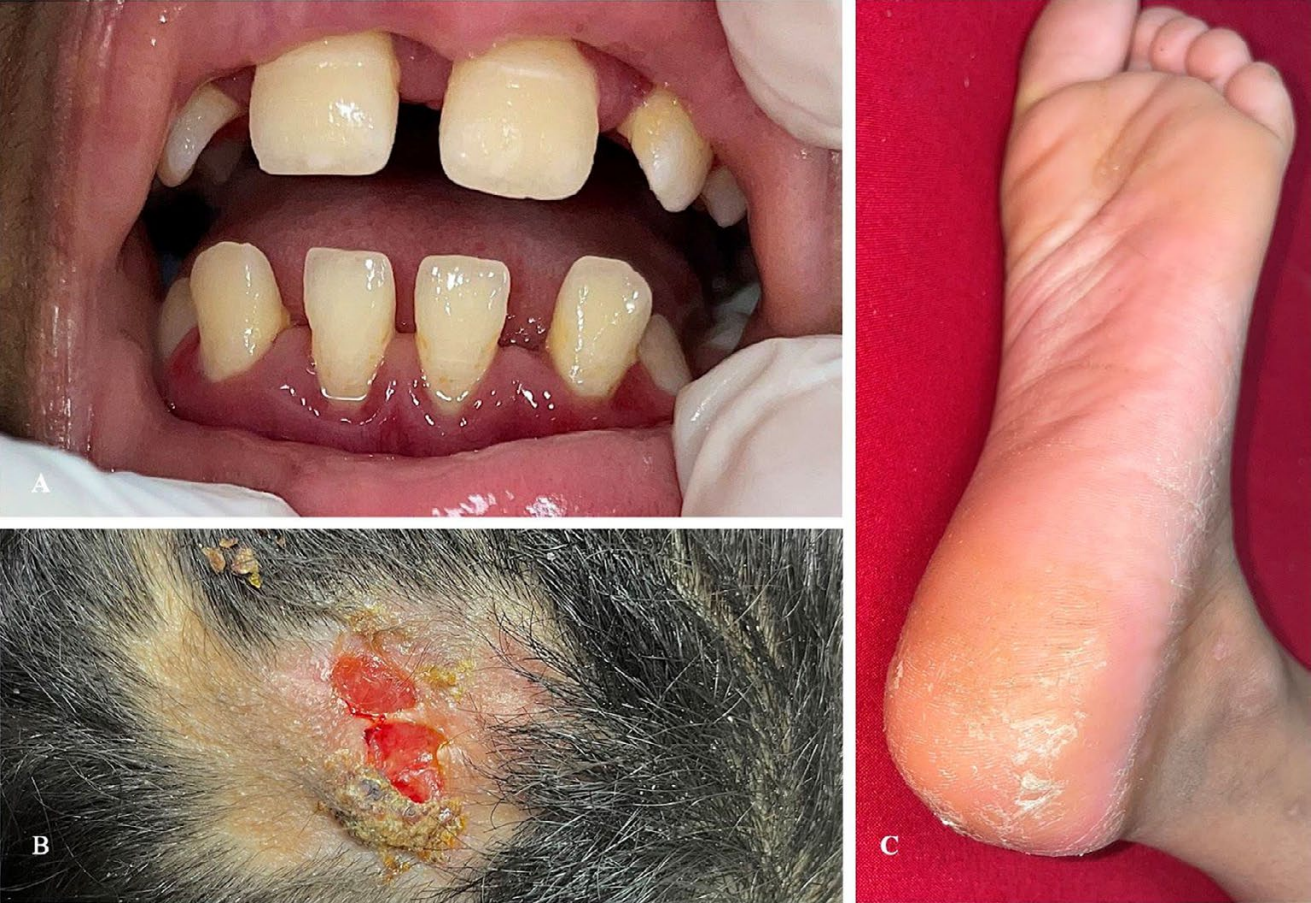
(A) Increased spacing of secondary dentition with signs of gingival inflammation. (B) Well‐demarcated ulcerated plaque with alopecia on the scalp. (C) Plantar erythema, hyperkeratosis, and scaling.

The patient's younger sister was examined, and the examination revealed plantar thickening and scaling, increased dental spacing with signs of gingival inflammation, and an ulcerated plaque on the scalp with alopecia. The alopecia was of the scarring type (Figure 7).

## 3 Methods

The biochemical and radiological workup was unremarkable. This included an ultrasound of the abdomen and pelvis, a skull X‐ray, and an X‐ray of the hands and feet to rule out liver and renal abscesses, intracranial calcifications, and acro‐osteolysis, respectively. A genetic analysis could not be done given the non‐availability of the facility in Pakistan. The workup did not identify features consistent with Nail and Patella syndrome.

## 4 Results and Conclusion

A clinical diagnosis of Papillon–Lefèvre Syndrome was established based on the patient's history and examination findings. The patient and her family received counseling regarding the diagnosis and its implications and prognosis. The treatment plan included oral Isotretinoin at 0.5 mg/kg/day for 2 months; after follow‐up, this dosage was continued for an additional 4 months. Additionally, the patient was prescribed co‐amoxiclav 625 mg TDS for 7 days and metronidazole 400 mg TDS for the same duration. In terms of topical treatments, calcipotriol 0.005% combined with betamethasone 0.05% was applied locally for 15 days, followed by calcipotriol 0.005% used twice daily for 2 months, along with paraffin‐based emollients plus urea 10% twice daily. The Surgery and Dentistry departments were involved in the management of the right big toe's granulation tissue and dental rehabilitation, respectively. Papillon–Lefèvre syndrome is a rare genodermatosis that presents significant morbidity. Consanguinity is recognized as a high‐risk factor. Early diagnosis and treatment can help mitigate morbidity in affected individuals, and raising societal awareness about consanguinity can contribute to reducing the disease burden in populations where this practice is prevalent.

## 5 Discussion

Despite the inability to pursue genetic testing for a confirmed diagnosis, the abundant clinical evidence in the patient, supported by the consanguinity of the parents and similar clinical findings in another sibling, helped in establishing the diagnosis of Papillon–Lefèvre Syndrome. Papillon–Lefèvre Syndrome is a rare autosomal recessive disorder whose prevalence is estimated at 1–4 cases per million individuals using statistical analysis [2]. The results of such estimations depend on the accuracy of the data on consanguinity in the study population, indicating that the mentioned prevalence may be an underestimation for populations with higher rates of consanguinity, such as Pakistan [11]. Higher numbers of PLS in countries with similarly high rates of consanguinity, for example, Saudi Arabia and India [12, 13], may serve as an indication of the prevalence of the disease in Pakistan. Early diagnosis and treatment may control the periodontitis observed in PLS and significantly delay or even stop the loss of teeth. Rigorous dental care under the supervision of a dentist, stringent oral hygiene, prophylactic antibiotic cover, and the use of oral retinoids have all been indicated in the preservation of teeth in PLS [14]. Dental prostheses can significantly reduce morbidity in patients with extensive teeth loss. Unfortunately, a timely diagnosis was missed in our patient, resulting in the loss of almost all teeth. Establishing the diagnosis in our patient helped us diagnose the younger sibling with PLS as well, allowing her to seek early treatment. The allelic variant of PLS, Haim–Munk syndrome, is extremely rare; however, cases have been reported in Pakistan and in Pakistani origin families [15]. Our patient had onychogryphosis of her toenails, a feature found in Haim–Munk syndrome [8], but lacked acro‐osteolysis and arachnodactyly. The remarkable finding noted in our patient was the presence of triangular lunulae, which are one of the characteristic features of Nail–Patella syndrome [16]. Although triangular lunulae can be identified in other circumstances, such as after nail trauma [17], our patient had no history of nail trauma or manipulation. To the best of our knowledge, literature on PLS has not mentioned the presence of triangular lunulae of nails, which in our patient indicates an extremely rare finding. The underlying mechanisms behind the development of the triangular lunulae remain an enigma; though its association with median nail dystrophy has been reported [18]. Identification of the triangular lunulae in our case opens the possibility of the feature occurring in other medical conditions as well.

## Author Contributions

**Zarak Khan Shiraz:** conceptualization, methodology, writing – original draft, writing – review and editing. **Fahad Faizullah:** investigation, writing – original draft, writing – review and editing. **Qaisar Ali Khan:** writing – original draft. **Irfan Ullah:** conceptualization, methodology, writing – review and editing. **Mehran Khan:** conceptualization, investigation, writing – review and editing. **Bader Semakieh:** writing – original draft. **Zain Ul Islam:** visualization, writing – original draft. **Ravina Verma:** writing – original draft.

## Consent

Written informed consent was obtained from the patient's parents for publishing this case report and its accompanying images.

## Conflicts of Interest

The authors declare no conflicts of interest.

## Data Availability

Data available on request from the authors.
